# Metabolic Hallmarks of Hepatic Stellate Cells in Liver Fibrosis

**DOI:** 10.3390/cells9010024

**Published:** 2019-12-20

**Authors:** Olga Khomich, Alexander V. Ivanov, Birke Bartosch

**Affiliations:** 1 INSERM, U1052, Cancer Research Center of Lyon (CRCL), Université de Lyon (UCBL1), CNRS UMR_5286, Centre Léon Bérard, CEDEX 03, 69424 Lyon, France; o.a.khomich@gmail.com; 2 Center for Precision Genome Editing and Genetic Technologies for Biomedicine, Engelhardt Institute of Molecular Biology, Russian Academy of Sciences, 119991 Moscow, Russia

**Keywords:** liver fibrosis, hepatic stellate cell, trans-differentiation, metabolism

## Abstract

Liver fibrosis is a regenerative process that occurs after injury. It is characterized by the deposition of connective tissue by specialized fibroblasts and concomitant proliferative responses. Chronic damage that stimulates fibrogenic processes in the long-term may result in the deposition of excess matrix tissue and impairment of liver functions. End-stage fibrosis is referred to as cirrhosis and predisposes strongly to the loss of liver functions (decompensation) and hepatocellular carcinoma. Liver fibrosis is a pathology common to a number of different chronic liver diseases, including alcoholic liver disease, non-alcoholic fatty liver disease, and viral hepatitis. The predominant cell type responsible for fibrogenesis is hepatic stellate cells (HSCs). In response to inflammatory stimuli or hepatocyte death, HSCs undergo trans-differentiation to myofibroblast-like cells. Recent evidence shows that metabolic alterations in HSCs are important for the trans-differentiation process and thus offer new possibilities for therapeutic interventions. The aim of this review is to summarize current knowledge of the metabolic changes that occur during HSC activation with a particular focus on the retinol and lipid metabolism, the central carbon metabolism, and associated redox or stress-related signaling pathways.

## 1. Introduction to Liver Fibrosis

Decompensation and hepatocellular carcinoma (HCC) due to liver cirrhosis are causing two million deaths every year, and account for more than 3.5% of all deaths worldwide [1]. HCC is the most common liver cancer and the second leading cause of all deaths related to cancer [2]. While the number of patients with liver disease has been steadily increasing over the last few years, treatment options for early, as well as late-stage liver fibrosis, remain limited. The most prevalent forms of fibrotic liver disease are alcoholic liver disease (ALD), non-alcoholic fatty liver disease, which frequently develops into non-alcoholic steatohepatitis (NASH) [3], and viral hepatitis. In all these cases, it is the chronic nature of the liver injury that drives fibrosis development. Fibrosis is a regenerative process that alters the homeostasis of the extracellular matrix (ECM) and leads on the long-term to excessive accumulation of connective tissue in the liver. Fibrosis is accompanied by immune infiltrations and altered angiogenesis. Together, these processes alter the architecture of the liver, reduce liver elasticity, and ultimately deteriorate organ structure and function [4]. Late-stage fibrosis, also termed cirrhosis, is characterized by the formation of nodules, where healthy liver tissue is ‘encapsulated’ by connective tissue. This results in liver stiffness and perturbation of blood flow with portal hypertension, and an increased risk of overall mortality and mortality due to liver decompensation or HCC.

Most of the progress that has been made in the identification of the molecular mechanisms underlying fibrosis is based on the use of in vitro model systems using tissue culture-adapted HSC lines, primary HSC preparations, and a number of in vivo models such as animals being fed high-fat/cholesterol or choline-deficient diets, animals that undergo bile duct ligation, treatment with ethanol, CCl_4_ or other chemical agents, or transgenic animals [5].

## 2. Hepatic Stellate Cells

**Figure 1:**
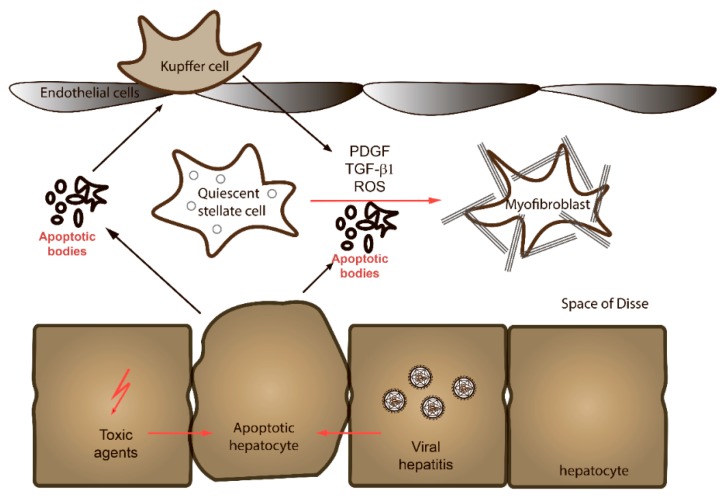
The space of Disse. Quiescent stellate cells located in the space of Disse undergo activation in response to a number of different stimuli, such as growth factors, cytokines or debris, and molecules derived from apoptotic hepatocytes. See text for details.

The cell type that is predominantly responsible for fibrotic processes is hepatic stellate cells (HSCs), a mesenchymal cell population that constitutes 5–10% of the total number of cells in the liver (Figure 1). HSCs are located in the perisinusoidal space (space of Disse) and are surrounded by hepatocytes and sinusoidal endothelial cells [6,7]. Their main functions are the secretion of laminin, proteoglycans, and type IV collagen to form basement membrane-like structures. Quiescent HSCs start to proliferate and undergo trans-differentiation into contractile myofibroblasts in response to paracrine stimulation by neighboring cell types, including Kupffer cells, hepatocytes, platelets, leukocytes, and sinusoidal endothelial cells. Kupffer cells can stimulate activation and proliferation of HSCs through the actions of cytokines, and in particular transforming growth factor β1 (TGFβ1), interleukin 1 (IL-1), tumor necrosis factor (TNF), reactive oxygen species (ROS) and lipid peroxides [6,8]. Hepatocytes are an important source of inflammatory lipid peroxides in liver diseases. Platelets release pro-fibrogenic growth factors such as platelet-derived growth factor (PDGF), TGFβ1, and epidermal growth factor (EGF). Neutrophils are an important source of ROS as well [6,8]. Lymphocytes represent a further potential source of pro-fibrogenic cytokines. Finally, HSCs are responsive to locally produced or systemic levels of vaso-constricting peptides and factors such as endothelin-1 and angiotensin-II. Endothelin-1 is produced primarily by the liver endothelium. Angiotensin-II is secreted by the liver and matured into a functional peptide by a number of proteolytic cleavages and nitric oxide to regulate the diameter of the sinusoidal lumen and thus the local microcirculation.

Upon stimulation, one major activity of myofibroblasts is to produce collagen fibers. Besides HSCs, liver progenitor cells and portal fibroblasts can also convert into myofibroblasts in response to pro-fibrogenic stimuli and contribute to collagen secretion.

Quiescent HSCs store retinoids (vitamin A) in lipid droplets in the cytoplasm [8]. Retinoid derivatives play an important role in tissue homeostasis and are implicated in proliferation, differentiation, and immune signaling [9]. Upon activation, HSCs lose the ability to store retinoids and start to proliferate, contract, and upregulate the synthesis of ECM components and ECM-modifying enzymes. Activated HSCs also produce pro-fibrogenic cytokines, growth factors, and morphogenetic proteins, which in turn regulate and impact tissue architecture. Expression of smooth muscle α-actin (α-SMA), the most prominent marker for identification of activated HSCs, alters the cellular cytoskeleton to drive cellular motility and contraction and is thought to regulate signaling processes during wound healing. The fibrogenic components produced by activated HSCs consist mainly of collagens type I and III, and ECM proteins such as, e.g., fibronectin, thrombospondin-1, and proteoglycans. Collagen, which consists predominantly of glycine, lysine, and proline, is first synthesized by HSCs as a pre-procollagen. Hydroxylation of amino acids, disulfide bonding, and glycosylation then occur within the endoplasmic reticulum (ER) to form a functional triple-helix collagen molecule. The collagen precursor is then secreted into the extracellular space, where peptidases cleave the amino-terminal and carboxy-terminal segments to produce insoluble collagen, which then forms microfibrils that combine to form a collagen fiber. Activated HSCs also express and regulate the activities of different families of ECM regulatory factors. Lysyl oxidase (LOX), lysyl oxidase-like proteins (LOXL), and transglutaminase are the predominant enzymes produced by HSCs that mediate crosslinking of collagen in the extracellular space [10,11,12,13]. The activities of matrix metalloproteases (MMPs), which degrade ECM at physiological pH, can be inhibited by HSC-expressed tissue inhibitors of metalloprotease (TIMPs) such as adamalysins (ADAMs and ADAMTS), meprins, and some others [14].

In the presence of persisting pro-fibrogenic triggers, due to chronic viral hepatitis or inflammation linked to metabolic syndrome, HSCs remain activated and, as a result, excessive ECM will accumulate over time, alter liver architecture, reduce the elasticity of the liver, impact blood flow through the liver, and increase the risk of organ failure and hepatocarcinogenesis. If the pro-fibrogenic triggers cease, fibrosis can disappear [15,16]. In respect to HSCs, they can either revert to a quiescent phenotype or undergo senescence or apoptosis [17]. Reversion of activated HSCs to a quiescent phenotype was, e.g., shown upon elimination of pro-fibrotic factors such as CCl_4_ or alcohol; however, the phenotype of HSCs remained distinct from that of quiescent cells. Expression of the fibrogenic genes collagen-α1, α-SMA, TGFβRI, and TIMP1 returned to baseline; however, expression of genes characteristic for quiescent HSCs such adiponectin receptor 1 and the lipid droplet binding factor perilipin 2 was not restored [18].

The observed changes in HSC phenotype during initiation and maintenance of fibrosis require metabolic alterations. This review focuses on metabolic mechanisms underlying fibrosis initiation and development.

## 3. Metabolic Alterations during HSC Activation

Recent analyses of the molecular mechanisms driving fibrosis have led to exciting new insights into the metabolic regulation of fibrosis. Cytokine signaling and contraction, as well as ECM synthesis and modification, require all metabolic reprogramming and adaptations that will be detailed below.

### 3.1. Retinol Metabolism

**Figure 2:**
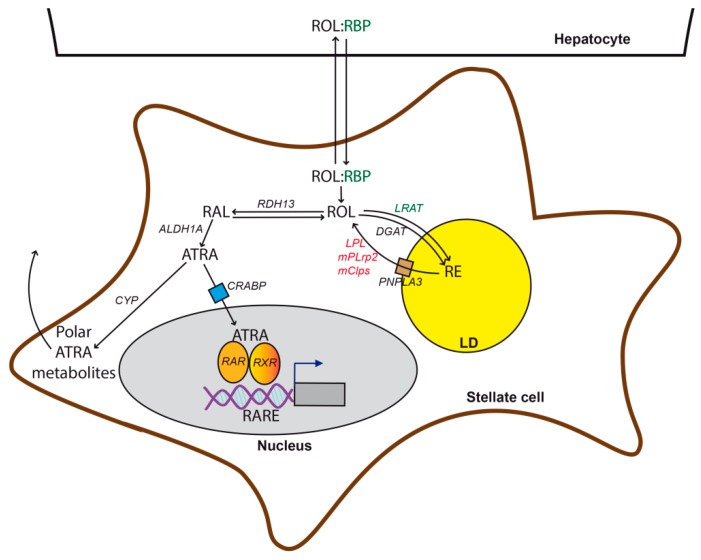
Scheme of retinol metabolism. Retinol (ROL) is imported from hepatocytes into HSCs being bound to retinol-binding protein (RBP). Upon its release, it is esterified by lecithin:retinol acyltransferase (LRAT) or diacylglycerol O-acyltransferase 1 (DGAT) and stored in lipid droplets (LD). Mobilization of retinol from lipid droplets is mediated by several enzymes including patatin-like phospholipase domain-containing protein 3 (PNPLA3), lipoprotein lipase (LPL), pancreatic-related protein 2 (mPlrp2), and procolipase (mClps). Retinol can be oxidized to retinal by retinol dehydrogenases (RDH), and the aldehyde can be oxidized to form all-trans retinoic acid (ATRA) by aldehyde dehydrogenase 1A (ALDH1A) isoforms. ATRA can be transferred to the nucleus when bound to Cellular retinoic acid-binding protein (CRABP), where it induces nuclear RA receptors (RARs) or retinoid X receptors (RXRs) to induce transcription of genes carrying retinoic acid response elements (RARE) in their promoters. Alternatively, ATRA can be metabolized by cytochrome P450 isoforms, and the resulting products are exported from cells. Genes that are up- or down-regulated during activation of HSCs are shown in red and green, respectively.

HSCs are the predominant cell type in the body that can store retinol or vitamin A (Figure 2). Retinol is required for vision, embryogenesis, cell differentiation, maintaining epithelia, and other functions. Retinol or its derivatives are absorbed in their hydrolyzed form (retinol) from the diet by enterocytes [19]. In enterocytes, retinol is esterified and the resulting retinyl ester is secreted and transported to different organs in chylomicrons [20]. Hepatocytes take up approximately 70% of total circulating retinyl esters. After hydrolysis in hepatocytes, retinol is transferred by retinol-binding protein (RBP) to HSCs for long-term storage [21,22]. There, retinol is converted into retinyl ester by lecithin:retinol acyltransferase (LRAT) and is stored in lipid droplets (LD) in the cytoplasm. Quiescent HSCs express high levels of LRAT and RBP. LDs in HSCs contain triglycerides, cholesterol, phospholipids, and free fatty acids [23]. Among them, triglycerides have the same abundance as retinyl esters. The number of LDs and their retinoid content are dependent on the dietary retinol intake. In response to low retinol content in other tissues, retinol is mobilized from HSCs. The first step of the mobilization is hydrolysis of retinyl esters to retinol with a subsequent transfer back to hepatocytes, where retinol is bound by RBP and secreted into the blood [24]. In plasma, the RBP-retinol complex is associated with transthyretin (TTR), and this association prevents elimination of the complex by the kidneys [25,26].

**Figure 3:**
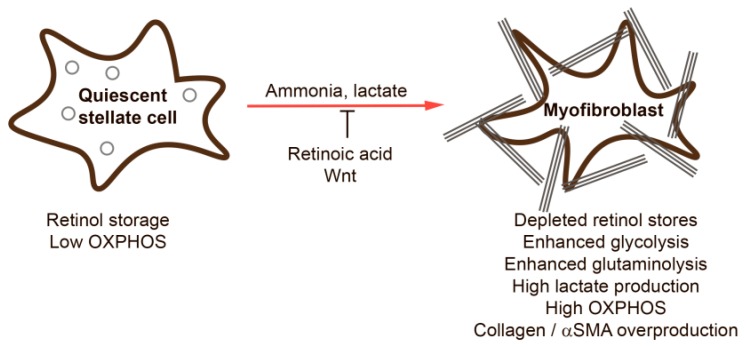
Features of quiescent and activated hepatic stellate cells. See text for details.

HSCs activation is accompanied by the loss of retinyl esters by mechanisms that are not fully understood (Figure 3). During HSC activation, expression of LRAT is significantly decreased [27], possibly via immune cell-mediated IL-1 signaling [28]. Curiously, LRAT deficiency is not associated with liver fibrosis; LRAT-deficient mice did not show any signs of liver fibrosis [27,29], and HCC development was delayed compared to controls upon injection of diethylnitrosamine [27]. On the other hand, administration of retinol or its derivatives ameliorated fibrosis and reduced CCl_4_-mediated oxidative stress in the liver [30] and was thus suggested as a potent anti-fibrotic treatment [31,32,33]. Similarly, dietary vitamin A deficiency is known to lead to fibrosis development and liver injury in rats [34]. While the molecular mechanisms by which loss of retinoic acids (RA) induce ECM accumulation are not well known, it is clear that inhibition of ECM synthesis occurs in response to RA treatment. The binding of RA to nuclear RA receptors (RARs) or retinoid X receptors (RXRs) has a suppressive effect on the promoter of alpha2(I) collagen [35,36]. In myeloid dendritic cells, vitamin A induces expression of MMP-9; however, the MMP-9 promoter does not contain a retinoic acid response element (RARE) and thus the underlying mechanisms of the increased expression of MMP-9 remain unknown [37]. Transgenic mice lacking RAR alpha, and thus having no RA signaling, develop steatohepatitis and liver tumors [38]. The influence of vitamin A deficiency and RA administration on ECM structure and content in different tissues are reviewed in detail in [39]. LRAT is the major enzyme responsible for retinol esterification, but diacylglycerol acyltransferase 1 (DGAT1) also possesses this function. In contrast to LRAT, inhibition of DGAT1 increases levels of retinol esters and reduces fibrosis markers in mice with NASH [40].

One of the enzymes responsible for retinol mobilization is patatin-like phospholipase domain-containing protein 3 (PNPLA3), which possesses triglyceride hydrolase, acetyl-CoA independent transacylase, and retinyl esterase activities [41]. Insulin and retinol regulate PNPLA3 expression. The PNPLA3 I148M mutation leads to the loss of enzymatic activity and subsequent changes in triglyceride metabolism and is associated with ALD, non-alcoholic fatty liver disease (NAFLD), and HCC [42]. The role of this mutation in the alterations in retinol metabolism is less clear. In line with the loss of enzymatic activity, retention of retinyl esters in HSCs and reduced plasma levels of retinol were reported [41,43,44,45]. However, it was also reported that expression of mutant PNPLA3 in HSCs leads to the depletion of retinol, but higher lipid droplet content was accompanied by increased release of proinflammatory cytokines, suggesting a role for this mutation in HSC activation [46]. Other enzymes probably involved in retinol mobilization are lipoprotein lipase (LPL), pancreatic-related protein 2 (mPlrp2), and procolipase (mClps), which were all reported to be significantly increased in activated HSCs [47,48]. Interestingly, in rat HSCs, seven days after activation of the culture by plating on a plastic surface, retinyl loss was not accompanied by triglyceride decrease [49]. Moreover, increased content of polyunsaturated fatty acids (FA) in LDs was observed.

An important question is if retinol loss in HSCs is due to increased mobilization or catabolism. According to existing data, LD components are mostly catabolized. Retinol dehydrogenase 13 (RDH13) catalyzes the first step of retinol catabolism—the oxidation to retinal. Recently, it was reported that RDH13-deficient mice exhibit reduced liver injury and fibrosis after treatment with CCl4, suggesting this enzyme as an important player in fibrosis [50]. The same data exist for alcohol dehydrogenase III (ADH3)-deficient mice [51]. In addition, it was shown that ADH3 reduces natural killer (NK) cell activity thus promoting HSC survival. Adipose triglyceride lipase (ATGL) also contributes to retinol degradation [52]. Another line of evidence that underlines the role of retinol catabolism in HSC activation is as follows: a metabolite produced during degradation, 9,13-di-*cis*-retinoic acid, stimulates fibrosis development via induction of TGFβ1 [53]. Another report showed that RA-mediated survival of B cells is required for liver fibrosis in response to CCl4 treatment, while B cell deficient mice did not develop fibrosis [54,55], suggesting that besides HSCs B cells are also important drivers of fibrosis.

Overall, the role of RA in fibrosis development is not very clear. RA administration has been reported to ameliorate the pathological process, while other reports suggest its pro-fibrogenic function [56]. Furthermore, several lines of evidence suggest that retinoic acids play a role in HSC senescence as described in the following review [17]. Briefly, exposure of activated HSCs to retinoids reverses their phenotype back to a quiescent state and promotes cell cycle arrest. Another mechanism of fibrosis reversal includes RA-mediated killing of early activated stellate cells by NK killers [57]. The induction of apoptosis in HSCs was suggested as a possible therapeutic approach [58].

### 3.2. Lipid Metabolism

Retinol metabolism is inseparable from the metabolism of lipids since retinol derivatives are present in the organism mostly in the form of palmityl or stearyl esters. Similar to treatment with retinoids, supplementation with palmitate suppresses HSC activation, mediated by LD-associated adipose differentiation-related protein (ADRP), and acts synergistically with retinol [59]. Loss of LDs is accompanied by changes in lipid metabolism. Several proteins associated with LDs regulate the lipid metabolism, such as, e.g., the perilipin (Plin) family. Activation of HSCs leads to the reduction of Plin5 levels, while Plin5 overexpression restores LD content and reverses HSC activation [60]. Liver fatty acid-binding protein (L-Fabp), which regulates FA metabolism, is inhibited during HSC activation; its overexpression leads to the restoration of LD content and attenuates expression of activation markers in cultured HSCs [61]. However, L-Fabp-deficient mice were less susceptible to steatosis and fibrosis in response to a high-fat diet [61]. In line with retinol administration, activation of lipid biosynthesis inhibits differentiation of stellate cells. Glycerol is a key molecule required for lipogenesis. Its transport into cells is mediated by aquaglyceroporins (AQPs), which are in turn regulated by antifibrogenic adiponectin [62]. HSC activation is characterized by a loss of retinoids from LDs. However, levels of triacylglycerol (TAG) species containing long polyunsaturated fatty acids (PUFAs) are increased in in vitro activated HSCs, an effect that is mediated by acyl-CoA synthetase type 4 (ACSL) [63]. However, in the same report, inhibition of ACSL reduced HSC activation, thus contradicting the previously mentioned positive effects of increased lipogenesis on fibrosis.

One of the key transcription factors that drive neo-lipogenesis is sterol regulatory element-binding protein (SREBP)-1c. SREBP-1c protects from HSC activation [64]. The hormone leptin, produced mainly by adipose cells for the regulation of body weight, reduces expression of SREBP-1c via p38 MAPK induction and liver X receptor inhibition. Leptin regulates FA metabolism and mobilization and thus plays an important role in liver fibrosis [65,66]. Interestingly, inhibition of NADPH oxidases, DPI, or knockout of neutrophil cytosol factor 1 (p47phox) significantly reduces leptin-mediated proliferation and differentiation of stellate cells [67]. Another link between lipid metabolism and fibrosis are liver X receptors (LXRs)—lipid-dependent nuclear receptors responsible for lipogenesis—which have an anti-fibrogenic role in HSCs [68]. LXRs form heterodimers with RXRs. Finally, the lipid sphingosine 1-phosphate (S1P) is known to stimulate HSC proliferation [69]. Regarding its role in the liver, it was reported that the S1P receptor is important for HSC proliferation in a CCl4 model of acute injury, but the authors mentioned it mainly for its role in tissue regeneration rather than in pathological processes [70]. Later, the impact of S1P signaling was shown in liver fibrosis development [71]. However, the exact molecular mechanism of S1P action during HSC activation is currently unknown. Other lipid mediators, such as autotaxin and its product lysophosphatidic acid (LPA), were suggested as blood markers for liver fibrosis progression [72,73]. At the same time, LPA itself acts as an inducer of HSC proliferation and contraction via Rho-kinase [74,75]. Recently, increased autotaxin expression in hepatocytes with subsequent accumulation of LPA in response to liver injury in mice was shown to lead to the activation of HSCs [76]. However, the exact roles of LPA in the metabolic processes associated with activation of stellate cells are not known.

Not much is known about the involvement of cholesterol metabolism in stellate cell activation. Disturbance of cholesterol metabolism in other liver resident cells types can lead indirectly to HSC activation. For example, in the case of NASH, an altered cholesterol metabolism is thought to activate Kupffer cells and trigger trans-differentiation of HSCs [77]. Regarding the direct effects of free cholesterol, it was reported that its accumulation in HSCs promotes fibrosis via TLR4 signaling [78], a process that is inhibited by acyl-CoA (cholesterol acyltransferase (ACAT1), which esterifies free cholesterol and thus prevents its accumulation [79]). Key regulators of the cholesterol balance are LXRs, and they furthermore link cholesterol and retinoid metabolism [80]. HSCs from Lxrαβ^−/−^ mice are characterized by the increased size of LDs due to accumulation of cholesterol and retinol esters. These cells are more sensitive to the activation signals and rapidly lose LDs under the regulation of associated protein Rab18 [80].

An interesting role in fibrogenesis plays the endocannabinoid system including arachidonic acid-based lipids (endocannabinoids) and their receptors, as well as enzymes responsible for their metabolism. Upon activation, HSCs accumulate cannabinoid receptor 2 (CB2), which is undetectable in a healthy liver [81]. This receptor has an anti-fibrogenic role. Moreover, the administration of endocannabinoids leads to specific death of hepatic stellate cells but not hepatocytes, thus revealing their anti-fibrogenic potential [82,83]. The underlying mechanisms were shown to be due to the expression of cyclooxygenase COX2 in HSCs, which converts the endogenous cannabinoid 2-arachidonoyl glycerol (2-AG) in pro-apoptotic prostaglandin glycerol esters [84]. In addition, cannabinoids have been shown to trigger ER stress-mediated apoptosis in activated HSCs, while quiescent HSCs and hepatocytes remained insensitive [85].

Excessive accumulation of lipids in hepatocytes, such as in the context of hepatic steatosis, can trigger HSC activation even in the absence of immune cells due to increased production of TGFβ and possibly other unidentified signaling molecules by hepatocytes [86,87]. Saturation of hepatocytes with lipids in NASH contributes to the production of lipotoxic compounds and cell death due to mitochondrial dysfunction and ROS production [88]. Finally, the treatment of hepatocytes with palmitic acid does not only induce apoptosis but also enhances the ability of hepatocyte-derived exosomes to activate HSCs [89].

### 3.3. Central Carbon and Nitrogen Metabolism

**Figure 4:**
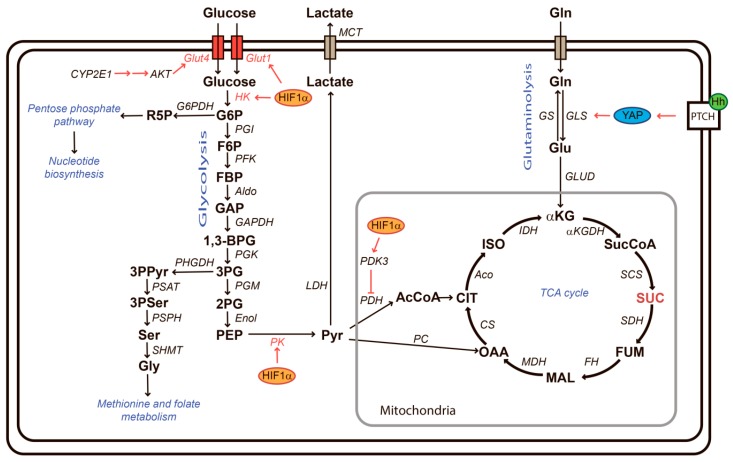
Central carbon metabolism. Glucose is taken up by glucose transporters (Glut) and then phosphorylated by hexokinase (HK) to produce glucose-6-phosphate (G6P). G6P can be channeled by glucose-6-phosphate dehydrogenase (G6PDH) into the pentose phosphate pathway for nucleotide production. Alternatively, G6P can be converted to fructose-6-phosphate (F6P), fructose-1,6-bisphosphate (FBP), glyceraldehyde 3-phosphate (GAP), 1,3-bisphosphoglycerate (1,3-BPG), and 3-phosphoglycerate (3PG) under catalysis of phosphoglucoisomerase (PGI), phosphofructokinase (PFK), aldolase (Aldo), glyceraldehyde dehydrogenase (GAPDH), and phosphoglycerokinase (PGK), respectively. 3PG is a precursor for de novo serine synthesis via 3-phosphopyruvate (3PPyr) and 3-phosphoserine (3PSer). Serine is converted to glycine to feed methionine and folate metabolism (one carbon metabolism). Serine/glycine biosynthesis is catalyzed by phosphoglycerate dehydrogenase (PHGDH), phosphoserine aminotransferase (PSAT), phosphoserine phosphatase (PSPH), and serine hydroxymethyltransferase 2 (SHMT2). Alternatively, 3PG is converted into pyruvate (PYR) via 2-phosphoglycerate (2PG) and phosphoenolpyruvate (PEP) with phosphoglyceromutase (PGM), enolase (Enol), and pyruvate kinase (PK) catalysis. Pyruvate is imported into mitochondria and either channeled to the tricarbonic acid (TCA) cycle via pyruvate dehydrogenase (PDH)-catalyzed formation of acetyl coenzyme A (AcCoA) or via pyruvate carboxylase (PC). Pyruvate can be also transformed into lactate through lactate dehydrogenase (LDH) and then be exported from a cell via monocarboxylate transporters (MCT). Glutamine can also serve as substrate for the TCA cycle. Glutaminolysis refers to the import of glutamine (Gln) and its conversion into glutamate (Glu) and α-ketoglutarate (αKG) through glutaminase (GLS) and glutamate dehydrogenase (GLUD). Within the TCA cycle, αKG is converted into succinyl-CoA (SucCoA), succinate (SUC), and fumarate (FUM) via α-ketoglutarate dehydrogenase (αKGDH), succinyl-CoA synthetase (SCS), and succinate dehydrogenase (SDH), respectively. Fumarate is converted into citrate (CIT) via malate (MAL) and oxaloacetate (OAA), and these reactions are accomplished by fumarase (FH), malate dehydrogenase (MDH), and citrate synthase (CS). Finally, citrate converted by aconitase (Aco) to isocytrate (ISO), and the latter is isomerized and then transformed into αKG with isocitrate dehydrogenase (IDH). Hypoxia-inducible factor 1α (HIF1α) upregulates expression of Glut1, HK2, PK, and PDK3, thus enhancing glycolysis but preventing shuttling of pyruvate into the TCA cycle. Cytochrome P450 2E1 (CYP2E1) activates a cascade (see the text for details) which leads to phosphorylation of alpha serine/threonine-protein kinase (AKT) and concomitant increase in Glut4 expression, thus also contributing to stimulated glycolysis. The Hedgehog (Hh)-YAP pathway upregulates expression of glutaminase 1 (GLS1). A TCA cycle intermediate succinate (also shown in red) may contribute to fibrogenesis by activating G protein-coupled receptor 91 (GPR91), also known as a succinate receptor.

Metabolic changes that occur in HSCs during activation share many common features with cancer cells. Cancer, but also viral infections, as well as immune cell activation, are characterized by strong cell proliferation and an increase of biosynthetic processes, as well as a balance between energy production, biosynthesis, and production of cytokines that modulate the extracellular environment [90]. Among these metabolic changes, the so-called Warburg effect in cancer cells is probably the best known and characterized by a flux of glucose that is converted away from a destination into mitochondria for ATP production toward intermediate metabolism for biosynthesis (Figure 4). The Warburg effect is frequently accompanied by a glutamine addiction. Glutamine-addicted cells require increased uptake of glutamine for amino acid exchange, a source of nitrogen for biosynthesis, for the maintenance of redox homeostasis, but also to feed the Krebs cycle in order to ensure the maintenance of ATP production [91]. In addition, changes to the amino acid and nucleotide metabolism, as well as the urea cycle, are frequently described in cells with high proliferation and biosynthetic rates.

The metabolic adaptations that HSCs undergo during activation and fibrogenesis have started to emerge over the last few years and predominant signaling pathways that mediate these metabolic adaptations include TGFβ, PI3K/AKT, MAPK but also Hedgehog (Hh), hypoxia-inducible factor 1α (HIF-1α) as well as Wnt signaling and downstream pathways.

HIF-1 α, stabilized by local hypoxia, oxidative stress or toxins, is an important pro-fibrotic actor, that induces not only the expression of pro-fibrogenic factors and enzymes such as PDGF, fibroblast growth factor, and plasminogen activator inhibitor-1, but also Lox1 and metalloproteinases, which in turn activate latent TGF-β1 [92]. Furthermore, as a master regulator of metabolic reprogramming, HIF-1α induces glycolysis as exemplified by upregulated expression of glucose transporter Glut1, hexokinase 2 (HK2), and pyruvate kinase as well as pyruvate dehydrogenase kinase 3 (Pdk3), which blocks the entrance of pyruvate into the Krebs cycle and thus favors conversion of pyruvate into lactate [93]. Accumulation of extracellular lactate produced by HSCs or transformed hepatocytes [94] may induce activation and metabolic reprogramming of neighboring hepatic stellate cells and may modify the immune environment, as demonstrated in breast cancer [95]. Furthermore, lactate accumulation is required for collagen synthesis and hydroxylation [96]. Glucose uptake has also been shown to be stimulated via increased expression of the glucose transporter GLUT4 in HSCs in a NASH model [97]. Mechanistically, this has been shown to be due to CYP2E1/leptin/purinergic receptor X7-mediated phosphorylation of AKT. Finally, high glucose in culture media of HSCs has been shown to trigger activation via the p38-MAPK cascade and ROS [98,99]. The fact that activation of glycolysis and lactate accumulation is a prerequisite for HSC trans-differentiation was demonstrated by treatment with Hh inhibitor GDC-0449 (targets smoothened) and the glycolytic inhibitor curcumin [93,100,101]. However, on the other hand, disruption of mitochondrial ATP production by prolonged, subtoxic doses of 3-bromopyruvate, even though accompanied by increased glycolysis and lactate production, were reported to be anti-fibrotic, suggesting that both glycolytic, as well as mitochondrial metabolic activity, are important for HSC activation [102].

In line with a requirement for mitochondrial metabolism, activation of glutaminolysis has been observed during HSC differentiation [103,104]. Hh mediates activation of the transcriptional co-activator Yes-associated protein YAP, which is essential for HSC differentiation into myofibroblasts [105,106,107]. Hh-mediated activation of YAP was shown to play an important role in the expression of glutaminase, a key enzyme of glutaminolysis, in HSCs [107]. Glutamine, converted to glutamate upon cellular uptake by the rate-limiting enzyme glutaminase, serves as a substrate for amino acid exchange, serves as a nitrogen donor for biosynthesis or the production of glutathione, and is converted into alpha-ketoglutarate to feed into the Krebs cycle for energy production. The importance of glutaminolysis in HSCs was demonstrated by the fact that their activation could be reversed with a pharmacological glutaminase inhibitor [107]. In addition, glutamine supports the respiration of activated HSCs [107]. Indeed, HSC activation leads to enhanced oxidative phosphorylation (OXPHOS) [108], a general feature of cells undergoing activation [109]. Inhibition of the Hh pathway with forskolin significantly reduced fibrosis development, oxidative stress, and inflammation in CCl_4_-treated rats [110]. In addition, inhibition of Hh signaling with Smo or Gli1 inhibitors reduced the expression of VEGF and angiopoietin 1 in HSCs [111].

Pointing to an important role for the amino acid metabolism, increased levels of glutamate in HSCs have been shown to result in the induction of 2-arachidonoglycerol (2-AG) synthesis, which in turn stimulates lipogenesis in hepatocytes [112]. Furthermore, the HSC activation marker α-SMA is partially dependent on the G protein-coupled receptor 91 (GPR91), also known as succinate receptor 1, which in turn is induced by succinate, an intermediate metabolite of the Krebs cycle. HSCs express the succinate receptor [113,114,115,116,117].

The Wnt signaling pathway was shown to suppress the activation of HSCs. Mechanistically, Wnt signaling mediates the induction of glutamine synthetase, an enzyme that mediates the reverse step of glutaminase and thus reduces levels of ammonia, a side product of the glutaminase reaction [118]. Ammonia is a metabolite that strongly activates HSCs [119]. However, contradictory data about the role of the Wnt pathway in liver fibrosis were also reported. Agonists of this pathway, roof plate-specific spondin (RSPO) proteins, are involved in HSC activation [120]. Moreover, the induction of Wnt signaling triggers HSC activation and loss of LDs by decreasing expression of RXR, RAR, peroxisome proliferator-activated receptor γ, and CCAAT/enhancer-binding protein α [121].

### 3.4. Redox Biology

Reactive oxygen and nitrogen species (ROS and RNS, respectively) produced by HSCs are key players in triggering HSC activation, proliferation, and apoptosis, and are either byproducts of the metabolic events coupled to HSC activation or produced by other enzymatic sources. Besides HSCs, damaged hepatocytes, Kupffer cells, or infiltrating lymphocytes also contribute to the activation and proliferation of HSCs as well as increased production of ECM via ROS/RNS production [122,123,124]. Interestingly, CCl4 treatment, one of the main models of liver injury and HSC activation, causes superoxide production in hepatocytes, which in turn stimulates HSCs as detailed in [125]. Several enzymes are known to stimulate fibrosis via impairing the redox balance [126,127]. TGFβ-induced apoptosis of hepatocytes is mediated by ROS and requires NADPH oxidase 4 (Nox4) upregulation [128]. The NADPH oxidase protein family consists of Nox1-5, Duox1, and Duox2 proteins. These enzymes transfer an electron from NADPH to oxygen with the generation of superoxide anion-radicals with the exception of Nox4 and Duox1/2, which produce hydrogen peroxide. Nox1 or Nox4-deficient mice show inhibited the development of CCl4-induced fibrosis, and Nox4 was also reported to be necessary for TGFβ-induced HSC activation and hepatocyte death [129,130,131]. Inhibition or knockout of Nox1 or Nox4 in cultured HSCs attenuates their activation and proliferation. Nox4 is upregulated in animals with CCl_4_-triggered fibrosis as well as in the liver of HCV-infected patients with virus-associated fibrosis. Induction of Nox1 and Nox4 occurs most likely in the context of the cascade TGFβ→Nox1→COX2→Nox4, as shown previously in Chang liver (CHL) cells treated with proinflammatory and toxic agents [132], and in Huh7.5 hepatoma cells expressing hepatitis C virus core protein [133].

Mechanistic studies showed that phagocytic NADPH oxidase Nox2^−/−^ mice represent less fibrosis in response to bile duct ligation or CCl_4_ [131,134]. Moreover, Nox2 was shown to be induced in HSCs isolated from fibrotic livers. A recent study revealed the role of Nox5 in the proliferation and activation of cultured HSCs (LX-2) [135]. Another enzyme, p66Shc, which regulates mitochondrial ROS production, was also shown to be involved in CCl_4_-induced fibrosis in mice and cultured HSCs [136]. p66Shc-associated increase of ROS levels leads to pro-fibrogenic inflammasome activation [137], suggesting an additional mechanism of ROS-mediated HSC induction. Migration of hepatic stellate cells, an important feature of mesenchymal cells, relies on activation of kinases ERK1/2 and JNK1/2 of the MAPK family in response to ROS production, pointing out another redox-dependent mechanism [138]. Another group found that ROS-mediated MMP-2 activation is important for HSC invasiveness, and is regulated by MAPK and PI3K pathways [139]. The pro-fibrogenic cytokine leptin also activates the MAPK pathway via ROS production, which was reported to be mediated by JAK [140]. Ethanol-inducible cytochrome P450-2E1 (CYP2E1), another important source of ROS, is implicated in the development of aging-related hepatic fibrosis in mice by causing nitroxidative stress [141]. Other fibrosis triggers, TGFβ and collagen type I, also stimulate stellate cell activation via redox signaling [142]. This signaling is mediated by NFκB, a classical redox-regulated factor, and transcription factor c-myb, and can be abrogated by antioxidant treatment. Therefore, an oxidative microenvironment in the liver is a common trigger for fibrosis development and progression.

The nuclear factor E2-related factor 2 (Nrf2) is a transcription factor that orchestrates the cellular antioxidant response by binding to the antioxidant response element (ARE) present in the promoter regions of a large set of ROS-scavenging genes. In HSCs, Nrf2 knock-down by RNA interference leads to activation and a more pronounced response to TGFβ1 pointing to the possible role of this defense pathway in the development of fibrosis [143]. This study was supported by various data showing an aggravation of liver injury in the absence of Nrf2 [144,145]; however, contradictory data have also been reported [146,147]. In respect to Nrf2 target genes, activated HSCs express, e.g., less SOD2 and catalase; however, glutathione content and glutathione peroxidase expression are induced [148]. In direct contradiction, expression of the catalytic subunit of glutamate–cysteine ligase (GCLC), another Nrf2 target gene that is required for glutathione synthesis, has been shown to decrease during activation of HSCs, while its upregulation may keep HSCs in a quiescent state [149]. Treatment of HSCs with all-trans retinoic acid (ATRA), which inhibits thioredoxin interacting protein and, therefore, elevates antioxidant activity of thioredoxin, reduces their activation [150]. A clinical study based on liver biopsies from 54 patients with non-alcoholic fatty liver disease (NAFLD) showed that the PNPLA3 I148M mutation, mentioned above, is accompanied by not only more pronounced fibrosis, but also increased oxidative stress [151]. The mechanistic link between this mutation and elevated serum markers of oxidative stress is currently unknown. Another important study revealed a strong link between immune reaction against lipid peroxidation products in serum and the risk of advanced fibrosis development in NAFLD patients [152]. Similar results were obtained in a mouse model of NASH [153]. Serum levels of nitric oxide metabolites correlated with the severity of autoimmune hepatitis and fibrosis [154]. The authors attributed it to the production of NO by immune cells.

Importantly, ROS can have a dual effect on HSCs. They can trigger their activation but also induce their apoptosis [125,155]. However, only activated but not quiescent HSCs seem to become apoptotic upon ROS exposure [156]. The authors suggested that the pro- versus anti-apoptotic effects of ROS on HSCs are linked to the presence of particular retinoic acids and differences in reduced glutathione levels. In contrast, another report showed that activated HSCs are resistant to superoxide-induced apoptosis, while low levels of superoxide production promoted cell migration and expression of several myofibroblast markers [157].

Death of hepatocytes and the resulting apoptotic bodies (AB) are known to activate HSCs indirectly via macrophages, but it has also been shown that stellate cells are capable to phagocytose AB themselves with subsequent induction of TGFβ [158]. Uptake of ABs by HSCs leads to overproduction of superoxide anion through induction of an unidentified member of the Nox family [159] and to stimulate HSC survival and activation by mechanisms involving JAK/STAT and NFκB signaling [160]. In murine NASH models leukocytes-derived myeloperoxidase (MPO) induces hepatocyte death and HSC activation via the production of an oxidant hypochlorous acid [161].

As mentioned above, activated HSCs exhibit enhanced OXPHOS phenotype [108], which is typically associated with increased production of ROS in mitochondria [109]. Unfortunately, there is no data showing how enhanced ROS production from various sources (at various sites within HSCs) triggers cell activation and trans-differentiation into myofibroblasts. This could be a goal of future studies carried out using various novel approaches of redox biology. In recent years, many lines of evidence point to the biological impact of ROS on enzymatic functions via modification of cysteine residues. A list of redox post-translational cysteine modifications includes oxidation into sulfenic and later into sulfinic and sulfonic acids, S-glutathionylation, nitration (nitrosylation), and others [162]. Proteins sensitive to ROS-mediated post-translational modifications are often referred to as redox switches. For the identification of such proteins, a set of approaches and tools have been developed over recent years [162,163]. Using specific probes, changes in HSC sulfenome/sulfinome during cell activation have been identified [164,165]. Besides identifying key redox-dependent players in fibrogenesis, these approaches may also allow estimating the input of various ROS sources into the fibrogenic process by analyzing their proximity to redox switches. Altered redox signaling is considered a promising target for fibrosis treatment by targeting, e.g., mitochondrial dysfunctions, and NADPH oxidases, as detailed in [166].

### 3.5. ER Stress

Fibrosis is associated with ER stress in biopsies of patients with different stages of fibrosis as well as in animal models of fibrosis induced by CCl4 or high-fat diet [167,168]. Moreover, induction of ER stress by tunicamycin treatment in mice triggered the expression of fibrosis markers (αSMA, TGFβ) in mice livers. The unfolded protein response (UPR) branch involved in fibrosis activation is protein kinase R (PKR)-like endoplasmic reticulum kinase (PERK), which indirectly induces SMAD2. In TGFβ-treated HSCs induction of another UPR branch, IRE1α occurs [169]. In line with this, treatment of cells with hydrogen peroxide or ethanol feeding *in vivo* led to UPR activation, while abrogation of the IRE1α branch of the UPR inhibited HSC activation and autophagy [170]. However, a recent report suggested that the induction of UPR during HSC activation is transient and not crucial for chronic fibrosis [171]. In this paper, tunicamycin did not induce activation of 3D cultured HSCs. The UPR has also been implied in the apoptosis of HSCs [172]. TGFβ-induced UPR was shown to activate transport and Golgi organization 1 (TANGO1), a protein required for collagen I secretion [173]. Loss of TANGO1 leads to UPR-mediated apoptosis of stellate cells and less hepatic fibrosis. These two controversial theories about pro- and anti-fibrogenic roles of ER stress seem to depend on the differential induction of the UPR branches and the timing of their induction and are discussed in the following review [174].

The PERK pathway of UPR triggers phosphorylation of eukaryotic transcription initiation factor 2α (eIF2α) thus blocking/attenuating cap-dependent translation. However, eIF2α can be also phosphorylated by three other protein kinases including general control non-depressible 2 (Gcn2). Gcn2 is primarily activated upon the accumulation of uncharged tRNAs [175], i.e., upon amino acid starvation. Although there are only scarce indications that amino acid pools are changed during HSC activation, activation of Gcn2 in primary or immortalized HSCs by withdrawal of the essential amino acid histidine suppressed collagen production with no detrimental effect on cell viability, suggesting that this enzyme plays an anti-fibrotic role in the liver [176]. The addition of exogenous leucine that should replenish amino acid starvation and resolve Gcn2 activation led to an enhancement of collagen alpha1(I) production pointing to an important role of this kinase in the regulation of HSC activation [177]. Both ER stress and Gcn2 activation can potentially lead to changes in amino acid metabolism. PERK/Gcn2-mediated eIF2α phosphorylation results in the induction of the transcription factor ATF4, which in turn controls the expression of an array of genes including asparagine synthase (ASNS), de novo serine biosynthetic enzymes [178], and several amino acid transporters [179]. However, these links need yet to be demonstrated in the context of liver fibrosis.

## 4. Conclusions and Future Perspectives

Liver fibrosis poses a worldwide health challenge due to its rising prevalence and concomitant lack of effective therapeutic strategies. A number of treatments that target the liver and in particular the glucose and lipid metabolism are currently undergoing clinical trials: FXRs regulate the metabolism of glucose, lipids, and bile acids. FXR agonists such as, e.g., obeticholic acid, ciofexor, tropifexor, and EDP 305 are undergoing clinical trials. Peroxisome proliferator-activated receptors are another nuclear receptor family involved in metabolic homeostasis and several agonists have/are being assessed in NASH patient cohorts. Furthermore, agonists of thyroid hormone receptor-beta signaling and inhibitors of the key lipogenic enzyme acetyl-coA carboxylase are being studied in patients. However, the overall efficacy of most of these drugs has been low. A detailed review of these clinical studies can be found in [180].

A more detailed understanding of the metabolic changes that HSCs undergo during the initial and chronic phases of fibrosis are highly important for the development of targeted intervention in order to reverse HSC activation or trigger HSC apoptosis. The similarities of the metabolic footprints of activated HSCs with that of cancer cells may be exploited in that respect. Indeed, in the cancer field, a number of pharmacological inhibitors targeting metabolic enzymes are becoming available for treatment and diagnosis [181]. However, the role of the metabolic microenvironment with local enrichment of metabolites is complicating therapeutic interventions. Indeed, nutrient availability, physical properties of the extracellular matrix, and interactions with stromal cells can all influence the metabolic phenotype of cancer cells and might ultimately dictate the response to metabolically targeted therapies [182]. Therefore, modulation of the metabolism of HSCs for therapeutic purposes needs to be carefully evaluated in the context of all liver resident cell types.
